# Continence Is Not Affected after Sling Revision with Transvaginal Tape Elongation for Post-Sling Voiding Dysfunction

**DOI:** 10.3390/jcm13020637

**Published:** 2024-01-22

**Authors:** Ching-Pei Tsai, Chih-Ku Liu, Evelyn Yang, Tsung-Ho Ying, Gin-Den Chen, Man-Jung Hung

**Affiliations:** 1 Department of Obstetrics and Gynecology, Taichung Veterans General Hospital, Taichung 407219, Taiwan; bariumym@gmail.com (C.-P.T.); mescal@vghtc.gov.tw (C.-K.L.); 2 Department of Obstetrics and Gynecology, Chung Shan Medical University Hospital, Taichung 402306, Taiwanying.steve@gmail.com (T.-H.Y.); gdchentw@hotmail.com (G.-D.C.); 3 Department of Obstetrics and Gynecology, School of Medicine, College of Medicine, Chung Shan Medical University, Taichung 402306, Taiwan

**Keywords:** bladder outlet obstruction, mid-urethral sling, surgical revision, urethral obstruction, urinary stress incontinence

## Abstract

Voiding dysfunction (VD) after sling operation is not uncommon. Sling revisions by incision/excision are usually effective; however, they may result in recurrent stress urinary incontinence (SUI). We aimed to evaluate continence status after an innovative sling revision procedure that preserves the integrity of the sling. Patients who underwent either a single-incision (AJUST) or a trans-obturator (TVT-O) mid-urethral sling were studied. Transvaginal tape elongation (i.e., sling midline incision and mesh interposition) was performed on patients with post-sling VD. Factors that may affect recurrent SUI were investigated by statistical analyses. Of 119 patients, 90 (75.6%) (45 AJUST and 45 TVT-O) were available for long-term (median 9; 8–10 years) follow-up. A significantly higher rate (17.2% vs. 3.3%, *p* = 0.014) of VD was noted after AJUST (N = 10) than after TVT-O (N = 2). After sling revision, four (33%) of the 12 cases reported recurrent SUI, which was not significantly different (*p* = 1.000) from the rate (37%, 29/78) of patients who did not undergo sling revision. Further statistical analyses revealed no significant predisposing factors affecting the recurrence of SUI. Surgical continence did not seem to be affected by having had sling revision with transvaginal tape elongation for post-sling VD.

## 1. Introduction

In the United States, the most common operation for the management of stress urinary incontinence (SUI) is sling surgery, including synthetic mid-urethral slings (MUS) or fascia pubovaginal slings [1]. Despite recent negative publicity regarding synthetic mesh use in vaginal surgery [2,3], numerous organizations support synthetic mesh use in anti-incontinence surgery [4]. Polypropylene mesh MUS is often recognized worldwide as the standard of care for SUI [5].

Voiding dysfunction (VD) after sling surgery is not uncommon. It has been estimated to occur in 2–25% of patients. Moreover, the incidence is likely to be under-reported due to patient satisfaction of being dry, and most patients who need sling revision seek a different surgeon. Post-sling VD causes a spectrum of symptoms ranging from irritative voiding to voiding difficulty with or without elevated post-void residual urine volume and total urinary retention [6,7,8,9,10]. In addition to these bothersome symptoms, delayed treatment may result in irreversible bladder dysfunction [11,12,13].

To date, no consensus has been reached regarding the diagnosis and treatment of this complication [6,7,8,9,10]. A postoperative urodynamic study did not seem to add much benefit in the clinical evaluation and prediction of outcomes [14,15]. Most surgeons rely on the temporal relationship between the operation and onset of associated symptoms to establish a diagnosis [6,7,8,9,10]. Initial conservative management includes catheterization, pharmacotherapy, and addressing circumstantial factors (e.g., pain) interfering with normal voiding [6,7,8,9,10]. Various sling loosening procedures performed at a short interval were reported to resolve the problem, and have been associated with minimal morbidity [10,16,17,18]. For refractory cases, sling revision with incision, excision of slings or extensive urethra-lysis are usually effective; however, these methods have been associated with risk of recurrent SUI in 9–61% of cases [6,7,8,9,10,13,19,20].

The aims of this study were to evaluate long-term surgical outcomes after two MUS procedures and subsequent sling revision using an innovative procedure (i.e., transvaginal tape elongation) for managing post-sling VD. Factors that may have affected the recurrence of SUI during the long-term follow-up period were investigated by statistical analyses. In this study, we hypothesized that sling revision with transvaginal tape elongation might help to resolve voiding symptoms while maintaining surgical continence, since the integrity of the sling is preserved.

## 2. Materials and Methods

### 2.1. Study Design

This was a retrospective cohort study conducted at a tertiary referral hospital. The surgical data of patients who underwent either a novel, single-incision (AJUST; C.R. Bard Inc., New Providence, NJ, USA) or a conventional, trans-obturator (TVT-O; Ethicon, Somerville, NJ, USA) MUS procedure for treatment of urodynamic SUI, between August 2013 and July 2015, were analyzed. All patients gave informed consent for one of the two MUS procedures after thorough counseling. The AJUST was performed on patients who requested it and were willing to pay an additional fee of about USD 1080 for the kit, which was not reimbursed by the National Health Insurance program in our country. For patients who did not want to pay this additional amount for treatment, TVT-O was performed. The exclusion criteria were previous continence surgery, concomitant gynecological and/or prolapse repair surgery, proven urodynamic voiding dysfunction, and diseases known to affect bladder or bowel function. Approval for this study was obtained from the Ethics Committee at our institution (CS2-21047).

### 2.2. Clinical Assessment

Pre- and post-operative assessment followed a standard protocol at our institution. In brief, at an outpatient clinic, each patient was interviewed using a validated symptom and quality-of-life questionnaire (i.e., UDI-6 and IIQ-7) [21] and underwent the following evaluations: a urinalysis, a pelvic examination, a cough stress test with a comfortably full bladder, and a uroflowmetry to measure the flow rate and residual urine. Follow-up examinations were performed postoperatively at 6 weeks, 3 months, 6 months, 12 months, and then annually to assess surgical efficacy and complications. A comprehensive multichannel urodynamic study was conducted pre-operatively, though it was not routinely repeated throughout the follow-up period.

The patients were ‘cured’ of SUI if they had a negative cough stress test result and there were no reports of urine leakage during stress (answer 0/not at all to UDI-6, Q3: leakage related to physical activity, coughing or sneezing). The patients who were considered to have ‘improved’ were those without leaks on the cough stress test, but they may still have had occasional urine leakage during stress. This occasional leakage, however, did not influence their daily activities. Patients who did not meet these criteria were considered to have ‘failed’.

The diagnosis of post-sling VD was made in patients who developed de novo symptoms consistent with voiding difficulties, persisting beyond the postoperative period. Non-invasive uroflowmetry tests showed an abnormal flow rate compared to preoperative levels, with or without elevated residual urine volume. Initial conservative treatments included pharmacotherapy and either intermittent or indwelling catheterization. If these measures were insufficient, a transurethral sling loosening procedure, as described by Karram et al. [16], was considered. This office-based procedure involves using a urethral sound to apply downward traction on the urethra, aiming to loosen the sling. In cases where the patient’s symptoms remained unresolved following these interventions, sling revision with transvaginal tape elongation was proposed.

### 2.3. Surgical Intervention

**Figure 1:**
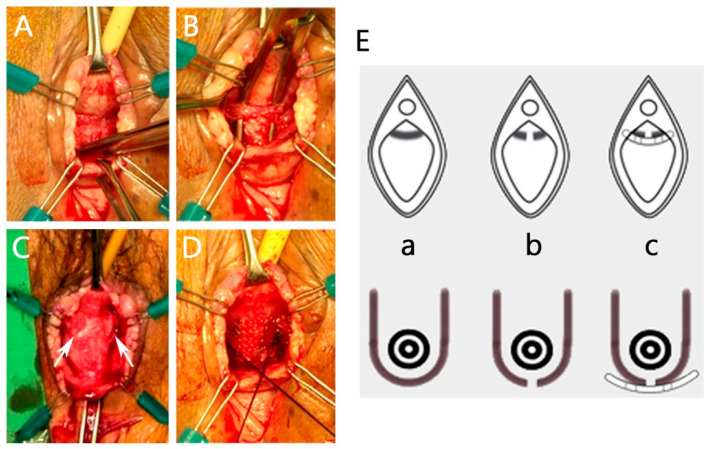
Sling revision with transvaginal tape elongation. (**A**) The sling is identified using careful sharp and blunt dissection. (**B**) A right-angle clamp is used to create a plane for sling incision. (**C**) The sling tension is released immediately after midline incision (cut edges labeled with white arrows). (**D**) A polypropylene mesh patch is used for elongation of the sling at middle urethra. (**E**) Illustrations of sling elongation procedures. (**a**) Before sling elongation. (**b**) Midline incision of the sling. (**c**) A polypropylene mesh patch is used for elongation of the sling.

The surgical team (C.-P.T. and M.-J.H.) was skilled in performing conventional (either retropubic or trans-obturator) MUS procedures. AJUST and TVT-O procedures were performed in a standard fashion according to the respective manufacturer’s instructions for use of the kits and video animations. A voiding trial began on postoperative day 1. In patients who were diagnosed with post-sling VD, sling revision with transvaginal tape elongation was performed as an outpatient surgery with the patients under intravenous general plus local anesthesia. The procedure was a modification of methods described by McLennan et al. [22], and its key steps are illustrated in Figure 1. This procedure is characterized by its minimal invasiveness and the preservation of sling integrity, mid-urethral position, and tension-free manner.

### 2.4. Statistical Analysis

SPSS for Windows version 15.0 (SPSS Inc., Chicago, IL, USA) was used for the statistical analysis. Descriptive statistics are presented as means ± standard deviation or percentages. Means were compared by unpaired *t*-test, and proportions were compared by Chi-square or Fisher’s exact tests, as appropriate. Univariate analysis was used to identify different variables between treatment groups. A multivariable logistic regression analysis was performed to investigate factors that may have affected surgical outcomes (continence vs. incontinence) at follow-up. All calculated *p* values were two-tailed, and *p* < 0.05 was considered statistically significant.

## 3. Results

### 3.1. Patients’ Characteristics

A total of 119 patients, who underwent either an AJUST (N = 58) or a TVT-O (N = 61) procedure between August 2013 and July 2015, were included in this study. Preoperative patient characteristics are shown in Table 1. Preoperative urodynamic study disclosed pure SUI with severe (one-hour pad test ≥ 10 g) degree of leakage in these patients. There were no statistically significant differences between the two groups regarding their general data.

### 3.2. Surgical Results

**Figure 2:**
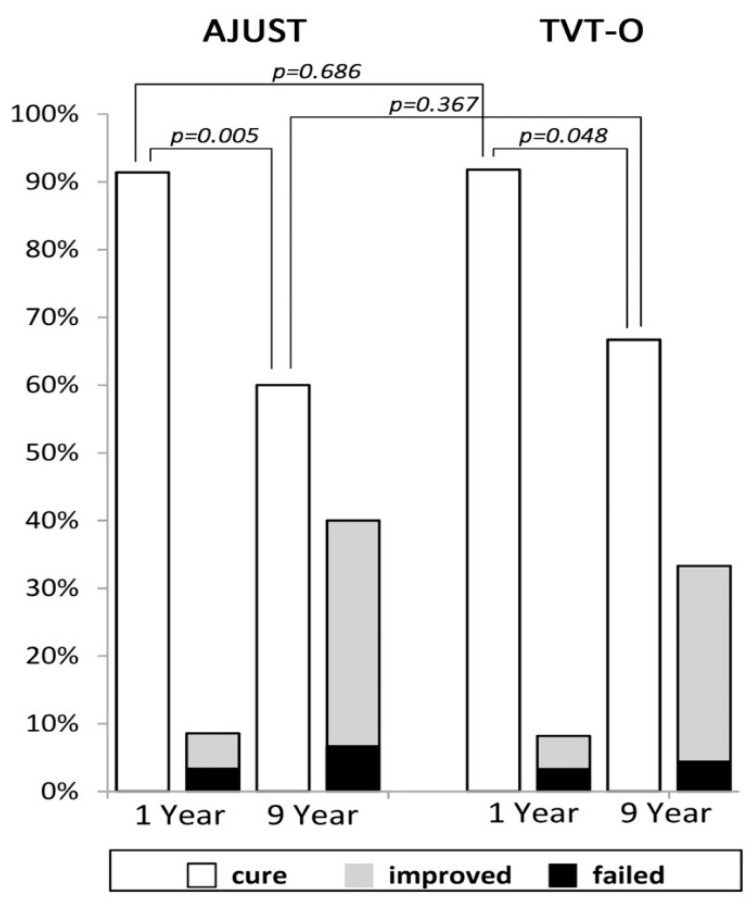
Surgical outcomes of stress urinary incontinence after AJUST and TVT-O at the postoperative one-year (N = 119) and nine-year (N = 90) follow-up.

All 119 (100%) patients attended the one-year follow-up, while 90 (75.6%) (45 AJUST and 45 TVT-O) of them were available for long-term (median 9; range 8–10 years) follow-up. The surgical results are summarized in Table 2. There were no statistically significant differences between the two groups with respect to the perioperative data and surgical outcomes, except for the significantly higher rates of VD after AJUST (N = 10) than after TVT-O (N = 2) (17.2 vs. 3.3%, *p* = 0.026). Post-sling VD was also the most prevalent (10.1%) complication in this study. Regarding surgical outcomes of SUI, continence (cure) rates after AJUST and TVTO declined significantly (*p* < 0.05) and similarly (*p* = 0.183) from one (91.4 vs. 91.8%) to nine (60.0% vs. 66.7%) years postoperatively. (Figure 2). UDI-6 and IIQ-7 scores at follow-up were also not significantly different between the two groups.

Symptoms, uroflowmetry, and surgical findings of the 12 patients who underwent sling revision for post-sling VD are listed in Table 3. The 12 (10 AJUST and 2 TVT-O) patients had all undergone a transurethral sling loosening procedure (out of 16 patients, i.e., 11 AJUST and 5 TVT-O) before sling revision. However, due to persistent voiding symptoms, they all agreed to receive revision of slings. Sling revision was performed at a median interval of 150 ± 141.2 (range 3–519) days from the primary surgery, with the majority (83%, N = 10) of them undergoing the procedure within 1 year postoperatively. In addition to undue sling tension, a distally displaced (either bilaterally or unilaterally) sling tape was found in seven (58.3%) of the twelve cases. Postoperatively, resumption of normal voiding was noted in all 12 (100%) cases; however, one (8.3%) and three (25%) patients, respectively, reported immediate and delayed recurrence of SUI at follow-up. The overall continence (cure) rate of this group of patients was 66.7% (8/12) at the long-term follow-up, which was not significantly different (*p* = 1.000) from the rate (62.8%, 49/78) found in patients who did not undergo a sling revision. Meanwhile, there were no procedure-related complications, such as persistent postoperative pain or vaginal mesh extrusion, in this group of patients.

### 3.3. Long-Term Continence Outcome

The overall continence (cure) rates after the two MUS procedures declined to 63.3% (57/90) at a median 9-year postoperative follow-up. A comparison of patients’ characteristics between those who were continent (cured) (63.3%, N = 57) or incontinent (36.7%, N = 33) at the long-term follow-up using univariate analyses is shown in Table 4. No statistically significant difference was found with the exception that incontinent patients reported significantly higher UDI-6 and IIQ-7scores, which indicated more bothersome urinary symptoms and poorer life quality. Further multivariable logistic regression analysis did not disclose any significant predisposing factors (i.e., age, body mass index, parity, abdominal leak point pressure, menopause, MUS procedures, follow-up interval, or having had sling revision or not) that may have affected the recurrence of SUI.

## 4. Discussion

In this study, we retrospectively reviewed patients with pure urodynamic SUI who underwent a single MUS procedure, either AJUST or TVT-O in our institution. Patients in the two groups had similar preoperative characteristics. By excluding confounding factors that may have interfered with the diagnosis of post-sling VD, the diagnosis was made mainly based on de novo symptoms suggestive of VD.

Post-sling VD was the most prevalent (10.1%) complication in this study, and AJUST contributed to this rate significantly more than TVT-O (17.2% vs. 3.3%, *p* = 0.026). However, the incidence decreased dramatically after the reduction of sling tension in later cases of AJUST, and VD no longer occurred after the 27th case in the AJUST group (N = 58). This finding suggests the existence of a learning curve for the performance of AJUST in this study. Spelzini et al. reported similar results when they used single-incision MUS as a new procedure for treatment of SUI [23]. In contrast to TVT-O, AJUST employs a non-deformable (vs. flexible) mesh fabrication and a strong obturator anchorage (vs. non-anchorage) [24,25], which may contribute to the higher obstruction rate when the same tension adjustment as that of TVT-O is used. Therefore, it is crucial to become thoroughly familiar with the biomechanical properties of a new MUS procedure before operation to avoid complications [26].

For cases of persistent VD following a sling procedure, revisions such as incising or excising the sling, or performing extensive urethralysis, are typically effective. Various mid-urethral sling incision methods, including midline, unilateral, and bilateral techniques, have been reported [6,7,8,9,10,13,19,20]. However, these approaches are associated with a 9–61% risk of SUI, as indicated in studies [6,7,8,9,10,13,19,20], which also lacked long-term follow-up. When the sling is incised or excised, particularly in early incisions, the area of the urethra above the sling gap becomes a weak point, increasing the vulnerability to incontinence recurrence. In our series, all patients experienced immediate improvement in LUTS following the sling elongation procedure. Although incontinence recurred in 4 out of 12 cases, 3 of these cases were mild and represented a significant improvement from pre-sling symptoms. Importantly, the recurrence of incontinence occurred over a longer period, not immediately following the sling elongation.

The clinical presentation with respect to symptoms and uroflowmetry varied greatly in the 12 patients diagnosed with post-sling VD in this study. Varied clinical presentations of urethral obstruction after anti-incontinence surgeries ranging from irritative voiding to total urinary retention have been reported [9,10,13,14,15]. However, urinary retention with a large amount of residual urine was rare in our patients, with only one (8.3%) of the 12 cases having residual urine volume greater than 100 mL. Therefore, VD of the 12 patients was thought to result from a partially obstructed MUS, because a transurethral sling loosening procedure had been performed before the revision. Initially, there were 16 cases (i.e., 11 AJUST and 5 TVTO) who underwent a sling loosening, and then one (9%) and three (60%) patients in the AJUST and TVT-O groups, respectively, were successfully relieved of voiding symptoms, obviating the need for sling revision. Notably, the effectiveness (9%) of the sling loosening procedure for AJUST was extremely low in this study, in contrast to the high success rates of 60% in this study and 80% reported by Karren et al. [16] when using the same technique in loosening a trans-obturator (i.e., TVT-O) and retropubic (i.e., TVT) MUS, respectively. The inferiority of this outcome may have resulted from the high-resistance properties of the AJUST procedure when compared to TVT-O and TVT [24,25]. Lo et al. also reported difficulty while trying to loosen the AJUST sling postoperatively [18].

During sling revision, a distally migrated sling was found in nearly 60% (N = 7) of the 12 patients in this study. Our findings are consistent with the results of a sonographic study conducted by Yang et al. They suggested a distally displaced MUS after implantation was more easily associated with obstruction, while a proximally displaced MUS was associated with recurrent/persistent SUI [27]. Currently, the exact mechanism behind sling migration is not fully understood. It is hypothesized that if the sling is overly tense, this might lead to its forward movement toward the region of fixation, such as the obturator foramen in mini-slings. A recent review of ultrasonographic findings of MUS after placement indicated that different sling tension and positioning related to the urethra may result in different surgical outcomes [28].

In addition to urethral obstruction, a distally migrated sling may also cause groin/thigh pain. In this study, two (3.4%) cases after AJUST (N = 58) who complained of unilateral groin/thigh pain had post-sling VD, and were found to have distally displaced sling at the ipsilateral side of the pain. Both pain and voiding symptoms subsided after revision of the slings. Theoretically, groin/thigh pain is a condition less associated with single-incision MUS than trans-obturator MUS, since the obturator foramen is not perforated [29]. However, the incidences of this complication were similar (*p* = 1.000) after AUST (3.4%) or TVT-O (4.9%) in this study. The mechanism of the complication after the two MUS procedures may be different. During sling revision, we found the distally displaced AJUST sling with undue tension caused traction and friction force on surrounding tissue, which may be a cause of the pain. However, symptoms subsided after sling revision with transvaginal tape elongation by which the integrity, middle urethral position, and tension-free manner of slings were restored.

Our results suggest sling revision with transvaginal tape elongation is a safe and effective method for treatment of post-sling VD without adding the risk of recurrent SUI at follow-up. After revision, four (33%) of the twelve patients reported recurrence of SUI, which was not significantly different from the rate (37%, 29/78) found in patients who did not undergo a revision. Further multivariate statistical analysis did not disclose any predisposing factors, including the sling revision that may affect the recurrence of SUI. Therefore, the decline in continence (cure) rates after the two MUS procedures and subsequent sling revision with follow-up was time dependent. Our results are consistent with findings from recent studies on various (i.e., retropubic, trans-obturator and sing-incision) MUS procedures, in that significant decline in continence was noted at a very long-term (≥10 years) follow-up [30,31,32]. Consequently, we suggest an aging process may develop postoperatively and act as a decompensating factor for the recurrence of SUI after MUS in some patients. This hypothesis is supported by another of our findings showing that patients with recurrent SUI also reported significantly higher UDI-6 and IIQ-7 scores with more stress and irritative (i.e., urinary frequency, urgency incontinence) symptoms than those who were continent at follow-up. Ageing is a well-known risk factor for the development of overactive bladder and mixed urinary incontinence [33,34].

The key strengths of this study are the homogeneity of the patient group and the long-term duration of follow-up. However, there were also some limitations. First, this was a retrospective study, and there was a relatively high rate (24.4%) of patients after MUS who were lost to the long-term follow-up. Second, there was no control group to evaluate the effects of sling incision without mesh interposition. Third, the AJUST sling was removed from the market in 2019. However, the results may be applicable to similar slings (a non-deformable mesh fabrication and a strong obturator anchorage) in the future.

In conclusion, the outcomes of long-term follow-up revealed that the continence (cure) rates after the two MUS procedures declined significantly and similarly, and did not seem to be affected by having had sling revision with transvaginal tape elongation for post-sling VD. Based on our results, we suggest the innovative sling revision procedure should be the method of choice for treating post-sling VD and the maintenance of surgical continence. However, further randomized control studies comparing sling incision with or without mesh interposition are needed to confirm the benefits of this procedure.

## Figures and Tables

**Table 1:** Comparison of preoperative characteristics of patients who underwent either an AJUST or a TVT-O procedure for treatment of urodynamic stress urinary incontinence.

	AJUST (N = 58)		TVT-O (N = 61)		
Characteristics	Value	Range	Value	Rage	*p* Value
General data					
Mean age (year)	54.4 ± 9.4	(37~82)	57.4 ± 12.1	(37~92)	0.296 *
Median parity	3	(1~5)	3	(1~4)	0.125 *
Mean body mass index (kg/m^2^)	24.6 ± 3.3	(19.4~33.7)	25.7 ± 3.5	(18.8–32.2)	0.225 *
% Menopause	56.9	(33/58)	59	(36/61)	0.705 #
% Diabetes mellitus	6.9	(4/58)	9.8	(6/61)	0.744 #
% Hypertension	24.1	(14/58)	31.1	(19/61)	0.420 #
% previous hysterectomy	8.6	(8/58)	18	(11/61)	0.620 #
Urodynamics (filling and voiding CMG)					
ALPP (cmH_2_O)	63.5 ± 19.1	(38~110)	64.3 ± 23.3	(28~135)	0.843 *
Cystometric capacity (mL)	314.5 ± 54.7	(199~410)	276.0 ± 102.8	(211~487)	0.938 *
Qmax (mL/min)	26.0 ± 10.4	(14~61)	21.0 ± 8.3	(12~38)	0.319 *
Qmean (mL/min)	11.5 ± 4.9	(6~30)	12.0 ± 4.5	(6~22)	0.460 *
Residual urine (mL)	14.6 ± 25.8	(0~100)	15.5 ± 28.4	(0~100)	0.976 *
One-hour Pad test (gm)	32.3 ± 14.8	(10~50)	33.9 ± 16.1	(10~50)	0.134 *

*: Mann–Whitney test; #: Fisher’s exact test. ALPP: abdominal leak point pressure. Qmax: maximum flow rate. Qmean: average flow rate. CMG: cystometrography.

**Table 2:** Surgical results of patients who underwent either an AJUST or a TVT-O procedure for treatment of urodynamic stress urinary incontinence.

	AJUST (N = 58)		TVT-O (N = 61)		
Patients’ Characteristics	Value	Range	Value	Rage	*p* Value
Perioperative data					
Mean hospital stays (days)	2.9 ± 1.1	(1~6)	2.6 ± 0.9	(1~4)	0.027 *
Mean total operating time (mins)	63.4 ± 14.4	(35~95)	63.2 ± 13.0	(45~85)	0.762 *
Mean estimated blood loss (mL)	71.1 ± 40.8	(50~150)	64.7 ± 22.3	(50~100)	0.874 *
Mean Foley drainage (days)	1.4 ± 0.8	(1~4)	1.2 ± 0.5	(1~3)	0.220 *
Mean PVR at post-op day 1 (mL)	107.1 ± 171.2	(0~800)	61.8 ± 66.5	(0~250)	0.468 *
Complications					
% Voiding dysfunction	17.2	(10/58)	3.0	(2/61)	0.026 #
% De novo UUI	3.4	(2/58)	4.9	(3/61)	1.000 #
% Groin/Thigh pain	3.4	(2/58)	4.9	(3/61)	1.000 #
Outcomes of SUI at 1-year					
% Cure	91.4	(53/58)	91.8	(56/61)	1.000 #
% Improvement	5.2	(3/58)	4.9	(3/61)	
% Failure	3.4	(2/58)	3.3	(2/61)	
Outcome of SUI at 9-year					
% Cure	60.0	(27/45)	66.7	(30/45)	0.317 #
% Improvement	33.3	(15/45)	26.7	(12/45)	
% Failure	6.7	(3/45)	6.7	(3/45)	
Outcomes of UDI-6 & IIQ-7 at 9-year					
UDI-6 total scores	1.78 ± 1.77	(0–5)	1.84 ± 2.46	(0–12)	0.744 *
Irritative scores	0.77 ± 0.87	(0–2)	0.67 ± 1.02	(0–4)	0.871 *
Stress scores	0.81 ± 0.93	(0–2)	0.69 ± 1.00	(0–4)	0.791 *
Obstructive scores	0.28 ± 0.45	(0–1)	0.49 ± 0.82	(0–4)	0.095 *
IIQ-7 scores	1.53 ± 2.76	(0–11)	1.80 ± 3.43	(0–12)	0.695 *

*: Mann-Whitney test; #: Fisher’s exact test. UUI: urgency urinary incontinence. UDI: Urinary Distress Inventory, IIQ: Incontinence Impact Questionnaire.

**Table 3:** Clinical presentation, uroflowmetry, surgical findings, and outcomes of the 12 patients who underwent sling revision with transvaginal tape elongation.

Cases by Slingorder	Sling Type- Series No.	Age	Symptoms	Q Max/Mean (mL/s)	RU	Days to Revision	Sling Position at Revision	Surgical Outcomes of SUI
01	AJUST-03	50	Frequency, groin/thigh pain	12/7	40	406	Distal (unilateral)	failure (immediate recurrence)
02	AJUST-07	49	Frequency, groin/thigh pain	38/21	60	519	Distal (unilateral)	cured
03	TVTO-07	56	Slow stream	10/4	30	254	Distal	cured
04	AJUST-08	67	Urgency	9/5	10	84	Middle	cured
05	AJUST-09	59	Slow stream, Multiple/positional voiding	2/2	40	147	Middle	cured
06	AJUST-14	51	Urgency	NA	40	80	Distal	cured
07	AJUST-15	63	multiple/positional voiding	7/3	Minimal	153	Distal (unilateral)	Improvement (delayed recurrence)
08	AJUST-18	52	Slow stream, recurrent UTI	31/9	Minimal	160	Proximal	Improvement (delayed recurrence)
09	AJUST-22	63	Frequency, slow stream	12/4	Minimal	267	Distal	Improvement (delayed recurrence)
10	AJUST-23	48	Frequency, urgency.	35/20	240	3	Middle	cured
11	AJUST-27	62	Urgency, urine stream deviation	19/8	40	22	Distal	cured
12	TVTO-28	48	Intermittent flow, groin/thigh pain	NA	Minimal	19	Middle	cured

Q: flow rates. RU: residual urine. NA: not available.

**Table 4:** Comparison of patient characteristics and symptom scores between continent (cured) and incontinent patients at a median 9-year postoperative follow-up.

	Continent (N = 57)		Incontinent (N = 33)		*p* Value
Age (years)	53.0	(48.0–63.0)	52.0	(45.0–60.5)	0.365
BMI (kg/M^2^)	24.7	(22.5–26.8)	23.4	(22.4–28.1)	0.719
Parity	3.0	(2.0–3.0)	2.0	(2.0–3.0)	0.462
ALPP (cmH_2_O)	58.5	(45.0–74.0)	62.0	(52.0–79.0)	0.285
Menopause	32	(56.1%)	16	(48.5%)	0.630
MUS procedures					0.662
% AJUST	27	(47.4%)	18	(54.5%)	
% TVT-O	30	(52.6%)	15	(45.5%)	
Sling Revision	8	(14.0%)	4	(12.1%)	0.382
Follow-up (years)	9.0	(8.0–10.0)	9.0	(8.0–10.0)	0.472
UDI-6 total scores at 9 years	0.93 ± 1.49	(0–7)	5.36 ± 3.87	(1–18)	<0.001
Irritative scores	0.44 ± 0.76	(0–2)	1.94 ± 1.84	(0–6)	<0.001
Stress scores	0.13 ± 0.34	(0–1)	2.61 ± 1.48	(1–6)	<0.001
Obstructive scores	0.36 ± 0.78	(0–2)	0.82 ± 1.31	(0–6)	0.077
IIQ-7 scores at 9 years	0.72 ± 2.03	(0–10)	4.00 ± 4.03	(0–12)	0.001

Chi-square test or Mann–Whitney U test. Median (IQR).

## Data Availability

The data presented in this study are available upon request after obtaining additional permission from the Institutional Review Board of Taichung Veterans General Hospital.
